# Supplementary material for: Learning curve and surgical outcome of robotic assisted colorectal surgery with ERAS program
Source: Sci Rep. 2022 Nov 29;12:20566. doi: 10.1038/s41598-022-24665-w (PMC9709162; doi:10.1038/s41598-022-24665-w)
Supplement: Supplementary file 2 — Supplementary Information 2. [file 41598_2022_24665_MOESM2_ESM.zip › RAL Raw data-Ñ[▒K/097/HS_ñw╝╨░O▒Kñσ.pdf]

正式摘要(電子簽章：000000221047249)

W92 / W92 ☐英文標題(注意:無英文姓名-請補病人基本檔!) ☐【讀取】憑證

臺中榮民總醫院

出院病歷摘要

轉入號： 轉出號：

[redacted]

(8)流水編號	
(9)入院日期	110/07/08 (2021) 直腸外科 W92 - 22
(10)轉科日期	
(11)出院日期	110/07/13 (2021) 住院天數計 5 日

診斷

- 入院 :A-colon cancer, 80cm FAV, s/p colonoscopic biopsy on 2021/06/19 at LMD
- 出院 :A-colon adenocarcinoma, 80cm FAV, s/p colonoscopic biopsy on 2021/06/19 at LMD, s/p Robotic RH on 2021/07/09, pT4aN2b(7/27)

主訴

- Suspected change of stool pattern for several months

病史

- This 43-year-old woman, without family history of colorectal cancer, denied abdominal surgery, was admitted for scheduled operation for maligant colon tumor.
  According to the patient, she denied significant symptoms, but suspected change of stool pattern for several months. There was no tarry stool, constipation or diarrhea, nor abdominal discomfort. There was no body weight loss or decreased appetite.
  She received her first colonoscopy during health check-up in 2021/06. At LMD, colonoscopy on 2021/06/19 showed suspected A-colon cancer, 80cm FAV, near obstruction. Thus, she was refered to our hospital for operation.
  Abdominal CT on 2021/06/29 showed a 6.2cm lesion, R/I ascending colon cancer with pericolic invasion and regional lymphadenopathy (cN2).
  This time, she was admitted for Robotic RH scheduled on 2021/07/09.

體檢發現

-
  - General appearance :
    - easy looking , conscious clear , without cardiopulmonary distress
    - Vital signs自動帶入date 體溫(BT) 脈搏(P) 呼吸(R) 血壓(BP) 身高(BH) 體重(BW) BMI
      20210708 36.2℃ 82次/分 18次/分 121/84mmHg 158cm 53.5kg 21.4
    - Vital signs: B.P. 121/84 mmHg , P.R. 82 /min , R.R. 18 /min , B.T. 36.2 ℃
  - HEENT :
    - Eyes: no anemic conjunctiva, no icteric sclera, normal EOM
    - ENT: no otorrhea, no rhinorrhea, no erythematous mucosa, no injected tonsil, no deformity
  - Neck: supple, freely movable, trachea midline,no deformity, no engorged jugular vein , no palpable thyroid or lymph node enlargement
  - Chest:
    - Inspection: Grossly normal, symmetric and fully expansion, no accessory muscle used, no operation scar, no spider angiomata, no gynecomastia
    - Palpation: no palpable mass or nodule, no subcutaneous emphysema, no tenderness
    - Ausculation: clear breathing sound
  - Heart:
    - Palpation: no thrill, no heave
    - Percussion: normal heart size
    - Ausculation: regular heart beat, normal S1, S2, no gallop, no murmur
  - Extremities: No deformity, full ranged of motion without limitation, no clubbing finger or nail cyanosis, no pitting edema, no ulcer or pigmentation, no obvious skin lesion, no varicose vein, no bone, joint or muscle tenderness

  CRS CONDITION

  - Karnofsky performance status: (100) Normal, no complaints, no evidence of disease
  - PE of Abdomen:
  - CEA: 1.70 ng/ml
  - Imaging Studies:
  - Colonoscope report: 80cm FAV, near obstruction (by LMD)
  - Sono , CT of abdomen and chest Films:Abdominal CT on 2021/06/29 showed a 6.2cm lesion, R/I ascending colon cancer with pericolic invasion and regional lymphadenopathy (cN2).
  - Figures:
    1. colon & rectum

手術日期及方法

- 手術日期及方法20210709 1. Robotic (XI) assisted right hemicolectomy
  2. Intraoperative colonoscopy : (-)
  3. Ileo-colic artery, right colic artery and right branch of middle colic artery were all ligated via robotic endoclips.

- 4. Take down from the hepatic flexure : (+)
  5. OpenGIA (80-3.8) X2 were applied to complete the side to side anastomosis
  6. Cut margin: proximal (ileum) 6 cm, (colon) 5 cm, distal 6 cm.
  7. penrose drain was placed at pelvis
  8. ADHESION BARRIER FILM was placed below the fascia of the para-umbilicus wound

住院治療經過

- After operation with Robotic RH on 2021/07/09, the patient was under post-operative care and pain control under PCA use. Self-urination was smoothly after removal of Foley's catheter on 7/10. The patient tolerated clear liquid diet and then Bland No.II diet on 7/12, No.III on 7/13, without N/V, abdominal pain or discomfort. The wound was clean and dry, without infectious signs. CVP was removed on 7/13. Under relative stable condition, the patient was discharge with OPD follow up.

合併症

一般檢查

- 累積報告 :

DATE	TIME	ALB	TP	BIL.T	BIL.D	ALKP	AST	ALT	LDH	AMY	LIPASE	NH3	Ferritin	UA
1100709	2043	3.4		0.4			21	14						
1100712	1214			0.26			19	11						

DATE	TIME	WBC	RBC	HGB	HCT	MCV	PLT	BLST	NEUT	BAND	SEG	LYM	MON	EOS	BAS
1100708	1310	6850		8.9	29.8	64.5	397								
1100709	2043	9650		8.2			334		91.4			7.2	1.3	0.0	0.1
1100712	1155	6060		7.4	25.0	68.1	300		80.0			14.7	4.8	0.3	0.2
1100713	0857			10.0											

DATE	TIME	NA	K	CL	BUN	CREAT	eGFR	CA	Mg	P	CRP	ESR	PCT	D-Dimer	LACTATE
1100708	1310					0.78									
1100709	2043	140	3.5		6	0.74									
1100712	1214	143	3.1		6	0.60	115.97								

DATE	TIME	GLUCOSE	HbA1C	CHOL	TG	HDL	LDL	KETONE	CK	CKMB	TRO-T	PT-P	APTT-P	NT-pro BNP
1100708	1626	76												
1100712	1143											11.2		

特殊檢查

病理報告

- 活體組織切片病理報告　手術日期：**110/07/09**　病理號：**S1021104**

診斷醫師:王任卿 報告日期:110/07/15
Pathologic diagnosis:
Ascending colon, terminal ileum, and appendix, right hemicolectomy
--- Adenocarcinoma, moderately differentiated, of ascending colon.
Tumor invaded to serosal surface and metastasized to mesocolic lymph nodes (7/27). The appendix, ileocecal valve and bilateral cut ends are free of tumor.
Ancillary study for diagnosis:
IHC stains for PMS2 and MSH6 are done on section G. Both above markers are preserved.
Prognostic and predictive factor:
1. Tumor size: 4x3.8 cm.
2. Depth of invasion: Visceral peritoneum (including tumor continuous with serosal surface through area of inflammation).
3. Histologic grade: G2: Moderately differentiated.
4. Angiolymphatic invasion: Present, extensive.
5. Perineural invasion: Present.
6. Tumor deposits (discontinuous extramural extension): Present, specify number of deposits: 7.
7. Macroscopic tumor perforation: Not identified.
8. Surgical Margins:
a) Proximal Margin: Uninvolved by invasive carcinoma, distance of tumor from margin: 8 cm.
b) Distal Margin: Uninvolved by invasive carcinoma, distance of tumor from margin: 7 cm.
c) Radial or Mesenteric Margin: Uninvolved by invasive carcinoma, distance of tumor from margin: 4 cm.
9. Lymph node status: Involved (7/27).
10. Extranodal involvement: Not identified.
11. Tumor regression grading S/P CCRT: No known presurgical therapy.
12. Type of polyp in which invasive carcinoma arose: Not identified.
13. Additional pathologic findings: Not identified.
14. Pathological TNM Stage: pT4aN2b (According to the eighth edition, American Joint Committee on Cancer Staging Guidelines for Tumors).
15. TNM descriptors: Not applicable.
Gross description:
The specimen consists of a segment of colon, 15 cm in length, and 4 cm in distal cut end circumference, and a segment of terminal ileum, 5 cm in length, and 2 cm in cut end circumference. An unremarkable appendix, 5.5 cm, is noted. A gray tan firm annular elevated and centrally ulcerative tumor, 4x3.8 cm, is noted, 7 cm to the proximal cut end. On cross section, the tumor invaded to the mesocolon with 40

- mm to the radial margin grossly. The appendix, ileocecal valve, and both cut ends are free of tumor. Representative part for section: A) proximal cut end B) distal cut end C) appendix D) ileocecal valve E-J) tumor K) radial margin L-N) lymph nodes. Blue ink indicated serosa; yellow ink indicated radial margin.
  Note:
  1. The interpretation of MMR panel and recommended studies are as below: (1) No loss of nuclear expression of MMR proteins: low probability of microsatellite instability-high (MSI-H) . (2) Loss of nuclear expression of MLH1 and PMS2: testing for methylation of the MLH1 promoter and/or mutation of BRAF is indicated (the presence of a BRAF V600E mutation and/or MLH1 methylation suggests that the tumor is sporadic and germline evaluation is probably not indicated;
  absence of both MLH1 methylation and of BRAF V600E mutation suggests the possibility of Lynch syndrome, and sequencing and/or large deletion/duplication testing of germline MLH1 may be indicated). (3) Loss of nuclear expression of MSH2 and MSH6: high probability of Lynch syndrome (sequencing and/or large deletion/duplication testing of germline MSH2 may be indicated, and, if negative, sequencing and/or large deletion/duplication testing of germline MSH6 may be indicated). (4) Loss of nuclear expression of MSH6 only: high probability of Lynch syndrome (sequencing and/or large deletion/duplication testing of germline MSH6 may be indicated). (5) Loss of nuclear expression of PMS2 only: high probability of Lynch syndrome (sequencing and/or large deletion/duplication testing of germline PMS2 may be indicated). (6) Please note: There are exceptions to the above IHC interpretations. These results should not be considered in isolation, and clinical correlation with genetic counseling is recommended to assess the need for germline testing.
  2. This case has been peer reviewed by two doctors.
  #T-59420_2 #M-81403_2 2 1105 S0000F
  #T-C4560_2 #M-81406_2 2 1105 000000
  #T-59200_2 #M-09183_2 0 1105 000000

其它

出院時情況

- 3.改門診治療

出院指示

藥名	劑量	劑量單位	用法	頻次	數量	數量單位	現況	天數	單位
Acetal tab 500mg	1	TAB	PO	BID&HS	21	TAB	TH	7	天
Flurbiprofen 100mg	1	TAB	PO	BIDPRN	14	TAB	TH	7	天
Mag. Oxide tab	2	TAB	PO	TIDPRN	42	TAB	TH	7	天
Through tab	1	TAB	PO	HSPRN	7	TAB	TH	7	天

出院計劃

- 門診日期
  - date on 2021/07/20

主治醫師 2062F 蔣鋒帆 / 住院醫師 2345B 陳映晴

正式摘要(**電子簽章：000000221047249)**

W92 / W92

doctorRank:B
